# Bovine leukemia virus *tax* gene/Tax protein polymorphism and its relation to Enzootic Bovine Leukosis

**DOI:** 10.1080/21505594.2019.1708051

**Published:** 2019-12-28

**Authors:** Irina M. Zyrianova, Svetlana N. Kovalchuk

**Affiliations:** Department of Molecular Biotechnology, Federal State Budget Scientific Institution Center of Experimental Embryology and Reproductive Biotechnologies, Moscow, Russian Federation

**Keywords:** Bovine leukemia virus (BLV), *tax* gene, Tax protein, polymorphism, Enzootic Bovine Leukosis (EBL)

## Abstract

Bovine leukemia virus (BLV) is an oncogenic retrovirus of the *Deltaretrovirus* genus, which causes persistent infection in its natural hosts – cattle, zebu, and water buffalo with diverse clinical manifestations through the defeat of B-cells. The BLV proviral genome, along with structural genes (*gag, pro, pol*, and *env*), includes nonstructural ones (*R3, G4, tax, rex, AS, pre-miRs* (for miRNAs). We have shown in our previous data the association of some *pre-miRs-B’* (for BLV miRNA) alleles with leukocyte (WBC – white blood cell) number in BLV-infected cows. Multifunctional properties of Tax protein have led us to an assumption that *tax* gene/Tax protein could have too population variations related to WBC counts. Here we report about several *tax* alleles/Tax protein variants, which have a highly significant association with an increase or a decrease of WBC number in BLV-infected cows. We have provided evidence that Tax A, H variants (*tax b, c, d, f, e* alleles) are correlated with reduced WBC counts at the level of BLV-negative groups of animals and thus could be the feature of the aleukemic (AL) form of BLV infection. We suggest this finding could be used in BLV testing for the presence of Tax A, H in the proviral DNA consider such strains of BLV as AL ones, and because of this, minimize the clinical losses due to BLV infection in cattle.

Bovine leukemia virus (BLV) with other three species: Primate T-lymphotropic virus 1, 2, 3 (PTLV-1, PTLV-2, PTLV-3, respectively), belongs to the *Deltaretrovirus* genus (*Orthoretrovirinae* subfamily, *Retroviridae* family, order *Ortervirales*) (the ICTV taxonomy database website: http://ictv.global/virus Taxonomy.asp).

BLV causes a persistent infection of B-cells in natural hosts – cattle, yak, zebu, and water buffalo resulting in the disease called Enzootic Bovine Leukosis (EBL). Two types of the disease manifestations can be distinguished: Persistent Lymphocytosis (PL) (about 30% infected animals) and B-cell leukemia/lymphoma (lymphosarcoma) or other types of tumors (< 5% ones). The remaining 70% of infected animals are asymptomatic and this is referred to as the aleukemic (AL) stage [1–5].

PL is a subclinical form of the BLV infection. It is a usually stable benign form of the disease with a nonmalignant polyclonal expansion of immature B-cells, which has been resulting in the increase of absolute WBC count (leukocytosis) in the peripheral blood [1,2]. Cattle with PL may suffer from disturbances of the immune system, and because of this could be susceptible to other infectious diseases (e.g. mastitis) [2,6]. The onset of PL usually occurs before the age of 5 years, and after that, PL may develop into the tumor stage (B-cell leukemia/lymphoma) with a peak of incidences between 5 and 8 years of cattle age, or may be not. As well the tumor stage could develop with or without prior PL [1,2,5].

So, it is difficult to say: is the PL type (form) or stage of the BLV infection disease? Some authors consider that only B-cell leukemia/lymphoma (lymphosarcoma) in the case of the BLV infection should be termed EBL [1]. Сlinical symptoms of tumors may manifest in digestive disturbances, weight loss, weakness. Superficial lymph nodes may be enlarged. A wide range of tissues and lymph nodes are found to be infiltrated by neoplastic cells, which can be detected at necropsy. Organs such as the abomasum, heart, spleen, intestine, liver, kidney, omasum, lung, and uterus are most commonly affected [5]. Experimentally, BLV can also infect rabbits, rats, chickens, pigs, guinea-pigs, cats, dogs, rhesus monkeys, chimpanzees, antelopes, goats, and sheep, but the only sheep can develop leukemia [5].

The BLV proviral genome has 8.7 kbp in length and includes structural genes (*gag, pro, pol*, and *env*) and non-structural ones (*R3, G4, tax, rex, AS, pre-miRs* (for miRNAs) [1,3,7–9].

In our previous data, we have shown an association of some *pre-miRs-B’* alleles with WBC counts in 2–5 yeas old cows [9]. Also, we have noted that a direct Tax-1 (of PTLV-1) and Drosha interaction has been found [10]. Drosha is one of the proteins participating in the biogenesis of host miRNAs [11], but it is not in the biogenesis of BLV miRNAs (miRs-B), which are processed without Drosha [12]. Tax 1 and Drosha interaction leads to the regular host miRNA production decrease [10]. The consequence of this is an increase of viral miRNA production. BLV Tax and host Drosha interaction has not been found, but it has been shown for miRs-B that their production increased so much, that BLV proviral DNA has been called “a prodigious producer of viral microRNAs” in host cells [7]. So, we have been suggesting that BLV Tax has interaction with Drosha too, and by this, it influences miRs-B’ production. Overall, there are much data that Tax (BLV Tax and Tax-1), except its primary function as a transactivator of proviral gene transcription, has essential roles in pathogenicity/oncogenicity. Tax involved in the transcriptional and posttranscriptional regulation of a wide variety of cellular genes. So, it takes part in many host cell processes: signal transduction, cell growth/proliferation/cycle, apoptosis, stress response, immunere sponse, DNA repair, and others [1,13–17]. So, having such multifunctional properties, the assumption about BLV Tax interaction with host cell Drosha is not illusive. Thereby, our suggestion about the effect of BLV Tax on miRs-B’ production could lead to *tax* gene/Tax protein polymorphism, and its association with WBC counts as it has been found for *pre-miRs-B’* genes [9].

In this study we have been investigating *tax* gene and Tax protein polymorphism, and its association with WBC counts. For this purpose, we have been used DNA from 42 peripheral blood samples of 2 to 5 years old female Holstein cattle (farming in Moscow region, Russia), all of which are the same as in previous works [9,18]. WBCs of the samples have been counted on an Abacus Junior Vet 5 Automatic Hematology Analyzer (Diatron, Austria).

All samples have been checked for BLV proviral DNA by high sensitive PCR test system based on two primers to *gag* and *pol* genes [19], and at the same time, all of them have been tested for antibodies to BLV envelope glycoprotein gp51 antigen by AGID (agar gel immunodiffusion).

The envelope glycoprotein gp51 is the main target for neutralizing antibodies in the host animal and is necessary for the virus to enter B-cells [5,8]. BLV antibodies can be detected by AGID, ELISA (enzyme-linked immunosorbent assay), RIA (radioimmunoassay), and immunoblot. These antibody detection methods, PCR detection of BLV proviral DNA, and BLV virus isolation are recommended to apply on the same clinical samples [5,8]. However, it should be noted, all of those methods reflect only the fact of a contact of an individual animal with the virus, but they do not allow predicting the spread of BLV infection to other susceptible cattle or development of tumors [18].

All of 42 samples have been divided into two groups: 16 BLV-seropositive and proviral BLV-positive ones (further: BLV-positive), and 26 BLV-seronegative and proviral BLV-negative (further: BLV-negative) ones and WBC count reference intervals (i.e. mean ± 2 SD) have been ranged as 4,710–29,322 and 5,906–16,609 WBCs/μL, respectively [9]. Further, 16 BLV-positive samples have been used to study *tax* gene/Tax protein polymorphism by PCR *tax* fragment cloning and sequencing. For this, two primers TBLV-F and TBLV-R (5ʹ-GCAAGTGTTGTTGGTTGGGGGCC-3ʹ and 5ʹ-CCCTCAAAAAAGGCGGGAGAGCC-3ʹ, respectively) have been designed based on the alignment of BLV complete genomic sequences retrieved from GeneBank used in our previous work [9]. (Primers had been synthesized by Evrogen JSC, Moscow, Russia.) Using these primers, ~ 900 bp PCR fragments of the second big exon of *tax* gene have been cloned with help of CloneJET PCR Cloning Kit (Thermo Scientific TM), and, further, the total of 83 clones (5–6 clones for each of 16 BLV-positive samples) have been sequenced by Sanger’s sequencing method (by Evrogen JSC, Moscow, Russia). The obtained sequences (GeneBank number from MN072344 to MN072355) have been aligned and analyzed with the GeneRunner program. We should note that the *tax* gene is presented in the provirus as composed of two exons, the first of which consists only of 4 bp. We have not found any substitutions in this short region after the alignment of complete genomic sequences of BLV from GeneBank. Because of this, we use only the second exon of *tax* gene, and further, when we are speaking about *tax* alleles and Tax protein variants, we consider only II-nd *tax* exon and Tax protein without Met and Ala, respectively, although we put them for comparisons with others from GeneBank.

As a result, we have detected twelve different *tax* alleles (*a* – *l*), which have many silent mutations. So, all *tax* alleles can represent eight variants of Tax protein (A – H). Moreover, all samples have been divided into three subgroups (I, I/II, II) with different representations of *tax* alleles/Tax protein variants (Table 1). I-st BLV-positive (BLV+ I) includes 6 cows (#1- 4,14,15) with *tax b, c, d, f* alleles, which represent Tax A protein, and *tax e* allele/Tax H protein. I/II-nd BLV-positive (BLV+ I/II) includes 7 cows (#5,6,9–11,13,16) with *tax a, l* alleles, which represent Tax B protein, and *tax j* allele/Tax G protein. II-nd BLVpositive (BLV+ II) includes 3 cows (#7,8,12) with *tax h* allele/Tax D protein, *tax i* allele/Tax C protein, *tax g* allele/Tax E protein, and *tax k* allele/Tax F protein (Table 1). Fisher’s exact test (http://mathworld.wolfram.com/FishersExactTest.html) has shown the highly significant (P < 0.01) association of such division of BLV-positive cows into three subgroups with different representations of Tax variants (and *tax* alleles). Namely, ones more, Tax A, H (*tax b, c, d, f, e* alleles) are present in the I-st BLV-positive subgroup, Tax B, G (*tax a, l, j* alleles) are present in the I/II-nd BLV-positive subgroup, and Tax D, C, E, F (t*ax h, i, g, k* alleles) are present in the II-nd BLV-positive subgroup (Table 1). WBC count reference intervals for BLV+ I, BLV+ I/II, and BLV+ II subgroups have been ranged as 8,009– 16,191, 7,097–30,540, and 10,140–35,146 WBCs/μL, respectively. We can see from ANOVA test (GraphPad Prism V.7.04, (1992–2017 GraphPad Software, Inc.) (Figure 1) and unpaired *t*-test (Table 2 (http://www. graphpad.com/quickcalcs/), that the difference between BLV+ I/II and BLV+ II subgroups has not been significant, so, it can be considered as one subgroup (Figure 1, Table 2). The opposite situation is with the BLV+ I subgroup. There is the significant difference in WBC counts of this subgroup with BLV+ I/II and BLV+ II subgroups. Moreover, the BLV+ I subgroup has not difference with the BLV-negative group. It means that the BLV+ I subgroup of cows remains AL. The same (with some exceptions) situation we had been found in our previous study for some of *pre-miRs-B’* alleles [9]. So, we can say that Tax A, H (*tax b, c, d, f, e* alleles) have represented in the BLV+ I subgroup could associate with the AL form (stage) of BLV infection, and these Tax (*tax* alleles) could be considered as AL ones. The rest Tax B, C, D, E, F, G (*tax a, g, h, i, j, k, l* alleles) have represented in the BLV+ I/II and BLV+ II subgroups could associate with the non-AL (PL or tumor) form (stage) of BLV infection.

**Table 1.** The occurrence of tax protein variants in each subgroup of cows: BLV+ I, BLV+ I/II and BLV+ II are BLV- positive subgroups of animals.

Tax alleles and Tax protein variants		The occurrence of Tax protein variants in each cow (in absolute numbers of 5–6 clones investigated) by subgroups															
		BLV+ I						BLV+ I/II							BLV+ II		
		1	2	3	4	14	15	16	13	5	6	9	10	11	8	7	12
*tax*	Tax																
*b, c, d, f*	*A*	6	5	5	4	6	5										
*e*	*H*				1												
*h*	*D*														4		
*i*	*C*														1		
*g*	*E*															5	
*k*	*F*																5
*a, l*	*B*							5	6	5	5	5	5	4			
*j*	*G*													1

**Table 2.** Unpaired *t-test* results for groups and subgroups of cows. The two-tailed *P* values with a 95% confidence interval. WBC RI (the WBC reference interval) has been measured in WBC/µL. BLV -, BLV +, BLV+ I, BLV+ I/II, and BLV+ II areas in Table 1 and Figure 1. BLV- and BLV + status has been determined by both serology (AGID) for BLV antibody, and PCR detection of proviral BLV DNA.

BLV – (N = 26) WBC RI is 5,906 ― 16,609				
*P* ≤ 0.0001 This difference is considered to be extremely statistically significant.	BLV + (N = 16) WBC RI is 4,710 ― 29,322			
	*P* = 0.1650 This difference is considered to be not statistically significant.	II BLV + (N = 3) WBC RI is 10,140 ― 35,146		
	*P* = 0.5195 This difference is considered to be not statistically significant.	*P* = 0.3797 This difference is considered to be not statistically significant.	I/II BLV + (N = 7) WBC RI is 7,097 ― 30,540	
*P* = 0.4768 This difference is considered to be not statistically significant.	*P* = 0.0730 This difference is considered to be not quite statistically significant.	*P* = 0.0054 This difference is considered to be very statistically significant.	*P* = 0.0223 This difference is considered to be statistically significant.	I BLV + (N = 6) WBC RI is 8,009 ― 16,191

**Figure 1.**
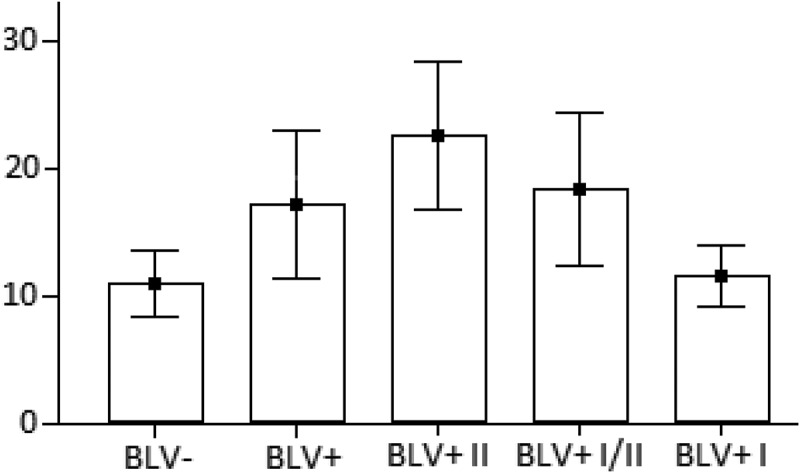
ANOVA test results for BLV-negative (BLV-), BLV-positive (BLV+) groups, I-st BLV-positive (BLV+ I), I/II-nd BLV-positive (BLV+ I/II) and II-nd BLV-positive (BLV+ II) subgroups of cows. The number of WBCs (y × 10^3^ cells/μL) is on the *y-*axis. BLV- and BLV+ status of these groups was determined by both serology (AGID) for BLV antibody, and PCR detection of proviral BLV DNA.

Furthermore, we have performed multiple alignments (Figure 2) and phylogenetic analysis (Figure 3) of Tax protein sequences obtained in this work and ones from GeneBank with known disease manifestations (Table 3) [4,8,20–25] using MEGA 10 program [26]. The evolutionary history had been inferred by using the Maximum Likelihood method and the JTT matrix-based model [27].

**Table 3.** Tax protein sequences have been used for the alignment (Figure 2) and the phylogenetic analysis (Figure 3). PL, T, AL, EBL*, EBL areas in Figure 2,3.

GeneBank numbers of Tax sequences				
different	the same	Genotypes	Manifestations of the disease	References
BAX04241.1		G1	EBL	[20]
CCJ67619.1		G1	T	[21]
BAX04259.1		G1	EBL	[20]
BAR47041.1	BAR47041.1	G?	T	[21]
	CCJ67631.1	G?	T	[21]
	CCJ67625.1	G?	T	[21]
	BAX04286.1	G1	EBL	[20]
	BAX04268.1	G1	EBL	[20]
	BAX04223.1	G1	EBL	[20]
	BAX04160.1	G1	AL	[20]
	BAX04151.1	G1	AL	[20]
	BAX04124.1	G1	AL	[20]
	BAX04115.1	G1	AL	[20]
	BAX04106.1	G1	AL	[20]
	BAX04088.1	G1	EBL*	[20]
	BAX04061.1	G1	AL	[20]
	BAX04052.1	G1	EBL*	[20]
	BAX47046.1	G?	T	[30]
BAP46819.1		G?	T	
BAX04169.1	BAX04169.1	G1	AL	[20]
	BAX04079.1	G1	AL	[20]
BAX04142.1		G1	AL	[20]
BAX04205.1	BAX04205.1	G1	EBL	[20]
	BAX04196.1	G1	EBL	[20]
	BAX04187.1	G1	EBL	[20]
BAX04277.1		G1	EBL	[20]
BAX04178.1		G1	EBL	[20]
BAU59290.1		G?	T	[22]
BAX04250.1	BAX04250.1	G1	EBL	[20]
	BAX04232.1	G1	EBL	[20]
	BAX04214.1	G1	EBL	[20]
BAX04133.1	BAX04133.1	G1	AL	[20]
	BAX04097.1	G1	AL	[20]
	BAX04070.1	G1	AL	[20]
AUV64408.1		G10	AL	[4]
ACR15161.1		G2	AL	[7,23]
AAF97920.1		G2	PL	[24,25]
AUV64420.1		G6	AL	[4]
AUV64414.1		G10	AL	[4]
AUV64426.1		G6	AL	[4]
AUV64438.1		G10	AL	[4]
AUV64432.1		G10	AL	[4]

**Figure 2.**
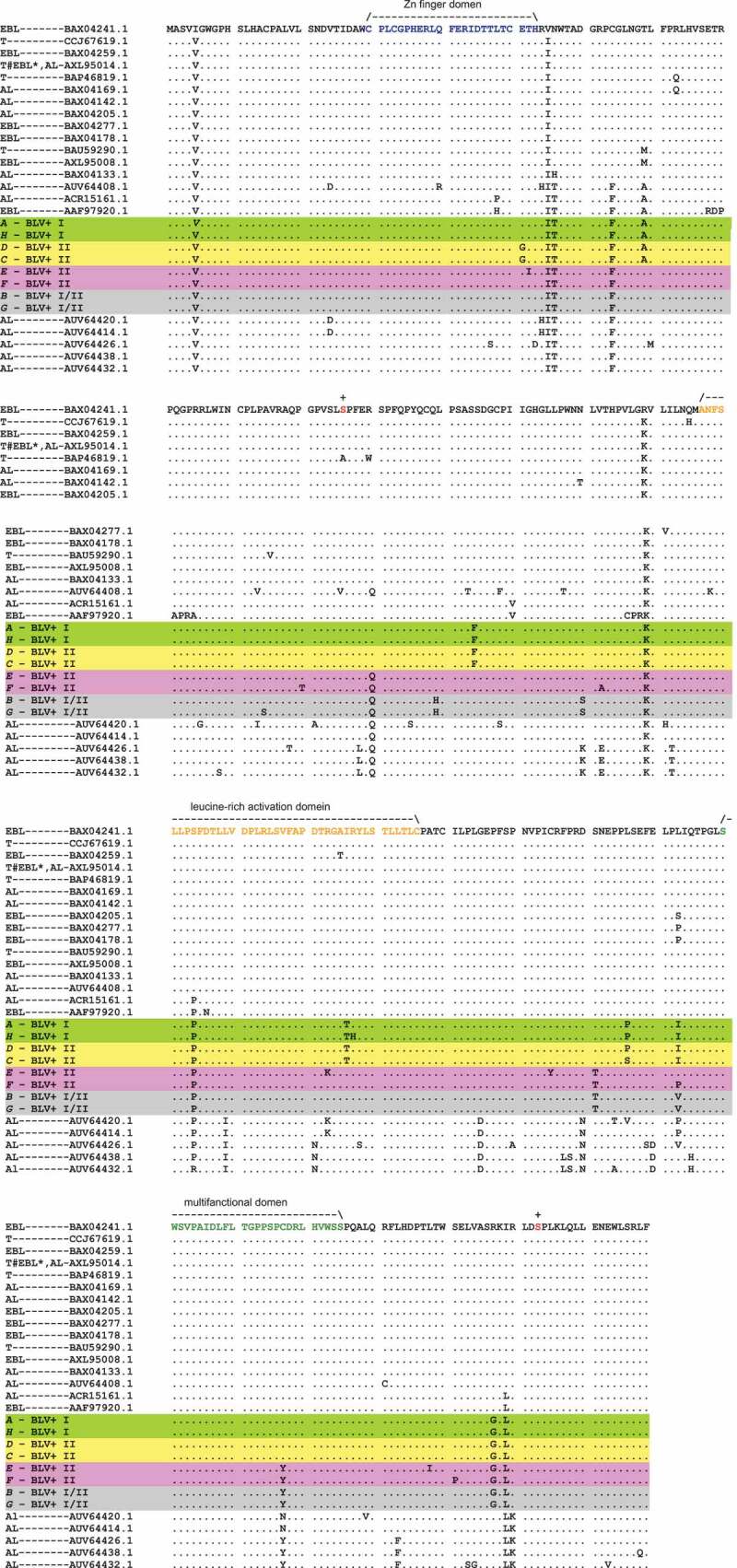
Alignment of Tax amino acid sequences./ – \― structural domains, + ― phosphorylation sites. BLV+ I, BLV+ I/II and BLV+ II areas in Figure 1. PL ― Persistent Lymphocytosis, T ― tumor (B-cell leukemia/lymphoma), AL ― aleukemic (asymptomatic, healthy or non-EBL), EBL* ― the disease has developed during the observation period (AL → EBL), EBL ― Enzootic Bovine Leukosis (PL or T are unknown) (Table 3).

**Figure 3.**
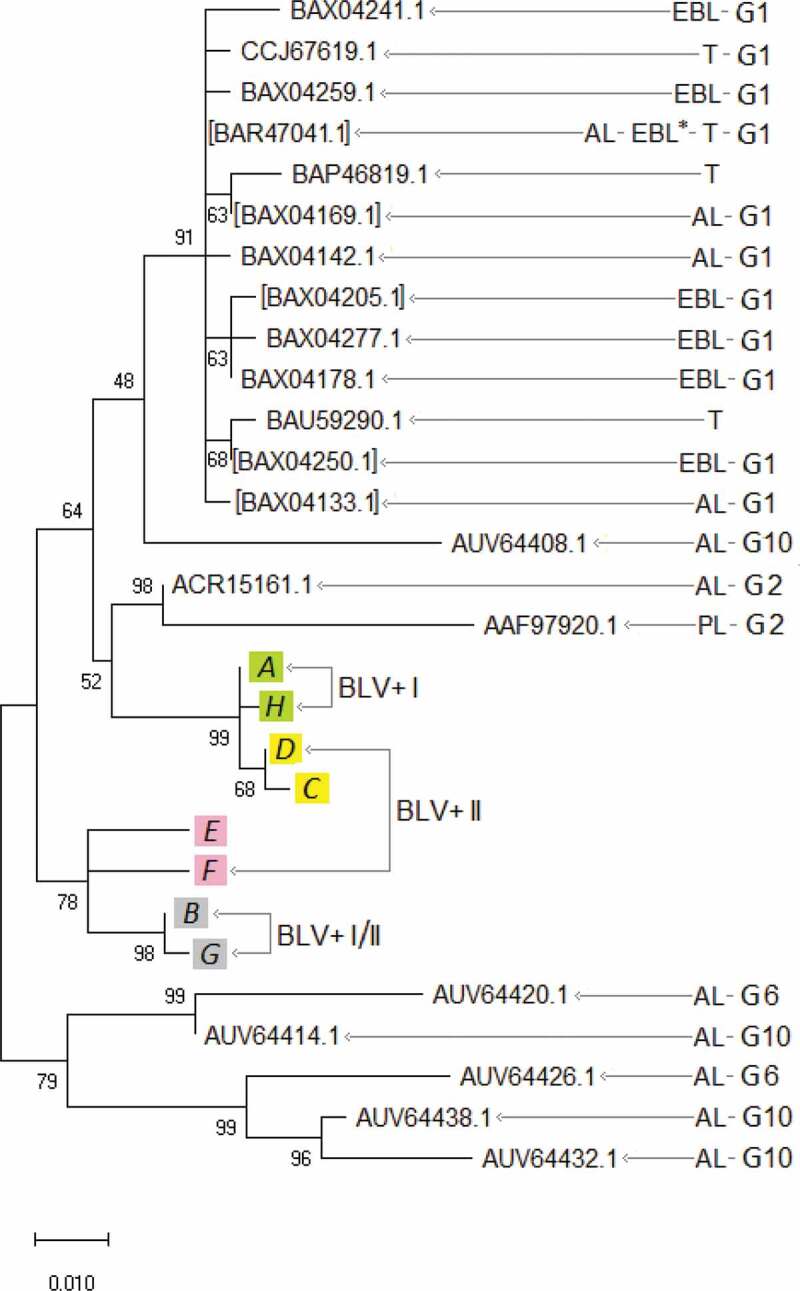
Tax primary protein sequence evolutionary analysis by Maximum Likelihood method. The percentage of trees in which the associated sequences clustered together is shown next to the branches. BLV+ I, BLV+ I/II, BLV+ II, PL, T, AL, EBL*, EBL areas in Figure 1, 2. G1, G2, G6, and G10 ― BLV genotypes, based on *env* genes and complete proviral genomic sequences, for which references are given in Table 3. Square brackets indicate the sequence numbers that represent groups of identical ones (Table 3).

We can see (Figure 3) that the Tax A, H (from the BLV+ I subgroup, AL ones) have clustered in the separate group from others. So, the AL form of the Tax has been separated in the phylogenetic tree from othes, which supposed to be non-AL (presented in BLV+ I/II and BLV+ II subgroups). A similar situation has been found for genotypes G1 and G2. The classification of BLV into ten genotypes (G1 – G10) has been made based on phylogenetic studies of *env* genes and proved by complete proviral genomic sequences [28]. In our phylogenetic analysis, based on Tax protein, the G1 and G2 phylogenetic groups (Figure 3) include BLV strains with AL and PL (for G2), and tumor (for G1) form (stage) of BLV-infection disease. We can, therefore, say that the AL or non-AL (PL or tumor) variants of Tax protein may be a refinement of BLV genotypes based on phylogenetic studies of *env* genes and complete proviral genomic sequences.

Farthemore, we can see (Figure 2) that the main difference between Tax A, H from Tax D, C is a mutation E51G in the putative zinc finger motif (ZNF) (Figure 2), one of the structural domains has been found in the Tax protein [14]. It is known that ZNF proteins participate in the regulation of a wide variety of genes and take part in diverse cell processes, such as DNA recognition, RNA packaging, transcriptional activation, apoptosis, cancer progression, and others [29]. Those properties of ZNF proteins remind the functions of the Tax proteins noted above. So, since the mutation E51G is located in the zinc finger (ZNF), it may be a key to different functionality of the Tax A, H (Tax E51) and Tax D, C (Tax G51) variants, which occur in different BLV+ subgroups (I and II, respectively) (Figure 1–3, Table 1), and I-st of which is considered as AL subgroup, and IInd does as non-AL one of the BLV+ animals (see above and Table 2).

In this study, we have discovered the existence of the *tax* gene/Tax protein polymorphism, which is related to the different WBC count in BLV-infected cows. We have provided evidence that Tax A, H variants (*tax b, c, d, f, e* alleles) are correlated with reduced WBC counts at the level of BLV-negative groups of animals and thus could be the feature of the AL form of BLV infection. So, we can suggest that BLV with Tax A, H variants may be considered as the AL strains of BLV. Moreover, this finding could be used in BLV testing for the presence of Tax A, H in their proviral DNA consider them as AL strains of BLV, and because of this, minimize the clinical losses due to BLV infection in cattle.
